# Invasive Candidiasis: Real-Time PCR-Based Species Detection and Antifungal Susceptibility Profiling of Suspected Patients in the ICU Using the Disc Diffusion Method

**DOI:** 10.7759/cureus.102182

**Published:** 2026-01-23

**Authors:** Shaila Akhtar, Raisa Badhan, Rafia Afreen Jalil, Mukesh Sharma, Md Zaber, Jannatul Nazerin Rubaiat, Sourav Debnath, Mahnaz Tabassum Raisa, Shaheda Anwar, Ahmed Abu Saleh

**Affiliations:** 1 Microbiology, Green Life Medical College, Dhaka, BGD; 2 Microbiology, National Institute of Burn and Plastic Surgery, Dhaka, BGD; 3 Microbiology, National Drug Control Laboratory, Dhaka, BGD; 4 Microbiology, Gazi Medical College, Khulna, Bangladesh, Khulna, BGD; 5 Microbiology, Kumudini Women’s Medical College and Hospital, Dhaka, BGD; 6 Microbiology, Dhaka Medical College, Dhaka, BGD; 7 Microbiology, Bangladesh Medical University, Dhaka, BGD

**Keywords:** antifungal susceptibility, candida species, disc diffusion, icu patients, invasive candidiasis, non-albicans candida, real-time pcr

## Abstract

Background

Invasive candidiasis (IC) is a major cause of morbidity and mortality in critically ill and immunocompromised patients, with a rising predominance of non-albicans *Candida *(NAC) species and increasing antifungal resistance. Rapid species identification and timely antifungal susceptibility testing are crucial for appropriate management.

Objective

To determine the species distribution of *Candida* in suspected ICU cases using real-time PCR and to assess antifungal susceptibility patterns by the disc diffusion method.

Methods

This cross-sectional study included 60 ICU patients with clinical suspicion of IC from September 2022 to August 2023. Clinical suspicion of invasive candidiasis was defined based on the presence of at least one of the following criteria: persistent fever unresponsive to broad-spectrum antibiotics, sepsis, or other systemic symptoms, such as hypotension, and relevant risk factors, including mechanical ventilation, central venous catheters, or prolonged use of broad-spectrum antibiotics. Blood samples were processed through automated culture, conventional microscopy, and species-level identification using multiplex real-time PCR targeting the ITS rDNA region. Antifungal susceptibility testing was performed using the Clinical & Laboratory Standards Institute (CLSI)-recommended disc diffusion method for fluconazole, itraconazole, voriconazole, and amphotericin B.

Results

Of 60 samples, 38 (63.3%) were PCR-positive for fungus. Among those, 24 were *Candida* species, and the remaining 14 were fungi other than *Candida* spp. The most common species detected was *Candida (C.) parapsilosis* (37.5%), followed by *C. albicans* (33.33%), *C. glabrata* (25%), and *C. krusei* (4.1%). Antifungal susceptibility testing revealed 100% susceptibility among *C. albicans* isolates to all tested agents. *C. glabrata* showed reduced susceptibility to fluconazole (50%), whereas *C. parapsilosis* demonstrated variable susceptibility across all antifungals. *C. krusei* showed complete resistance to all azoles but remained sensitive to amphotericin B.

Conclusion

NAC species predominated among ICU patients with suspected invasive candidiasis and exhibited higher resistance rates, especially to azoles. Real-time PCR provided rapid and accurate species identification, while disc diffusion offered reliable antifungal susceptibility profiling. Integrating molecular diagnostics with routine susceptibility testing is essential for guiding timely, targeted therapy and improving clinical outcomes in critically ill patients.

## Introduction

Invasive candidiasis (IC) is a life-threatening fungal infection, particularly among hospitalised, critically ill, and immunocompromised patients. Although *Candida (C.) albicans* was historically the most common cause, recent global trends show a shift toward non-albicans *Candida* (NAC) species such as *C. glabrata*, *C. tropicalis*, *C. parapsilosis*, and the emerging *C. auris* [1-3]. This shift is clinically significant because different species vary in their virulence factors and antifungal susceptibility, which, in turn, influence treatment outcomes [1-3].

Accurate and rapid species-level identification is essential for effective management of invasive candidiasis. Conventional culture-based methods, including germ-tube tests and chromogenic agar, are often slow and sometimes unreliable for differentiating closely related species [4]. Molecular methods, such as real-time PCR (RT-PCR), provide a faster and more sensitive alternative, enabling the direct detection and precise discrimination of clinically relevant *Candida* species from patient specimens [4,5]. The use of multiplex RT-PCR assays that can detect major *Candida* species within hours has significantly improved early diagnosis and guided targeted antifungal therapy [5].

Antifungal susceptibility testing (AFST) is another crucial component of IC management. Rising resistance to azoles, especially fluconazole, has been documented worldwide, particularly among NAC species [6]. Disk diffusion remains one of the most widely used phenotypic methods in clinical microbiology settings because it is simple, cost-effective, and aligned with Clinical & Laboratory Standards Institute (CLSI) recommendations [6,7]. Several studies have shown good concordance between the disk diffusion and reference dilution methods for commonly used antifungals [8].

In Bangladesh, invasive *Candida* infections are increasingly recognised, with a growing burden of NAC species. A tertiary-care study from Dhaka reported 109 *Candida *isolates, of which only 37% were *C. albicans*, while NAC species, such as *C. parapsilosis*, *C. ciferrii*, *C. tropicalis*, and *C. glabrata,* predominated; importantly, C. auris was also detected in bloodstream samples [3]. Fluconazole resistance was significantly higher among NAC (29%) than* C. albicans* (10%), while overall susceptibility to voriconazole remained high (~95%) [3].

Another study from an intensive care unit in Mymensingh reported an alarming predominance of NAC in candidemia cases, 89% of 39 isolates with *C. parapsilosis* (41%), *C. ciferrii* (23%), *C. auris *(7.7%), and *C. rugosa* (10.3%) identified [9]. Approximately one-third of isolates showed resistance to fluconazole, highlighting the emerging antifungal resistance problem in the country [9].

Bangladeshi neonatal units have also reported substantial NAC involvement in sepsis. A NICU-based study in Dhaka found that 39.7% of neonatal sepsis cases were caused by NAC, predominantly *C. tropicalis* (81%). Alarmingly, fluconazole susceptibility was extremely low (~3.5%), while voriconazole susceptibility remained high (98.3%) [10]. Additional reports have documented the emergence of *C. auris* and other uncommon species in clinical samples in Bangladesh [11,12].

These findings underscore the increasing species diversity and antifungal resistance among *Candida* isolates in Bangladesh. Despite this, integration of rapid molecular species identification with phenotypic susceptibility testing is still limited. Therefore, the present study utilizes real-time PCR for precise species identification in invasive candidiasis and complements it with disk diffusion antifungal susceptibility profiling. This study aimed to identify *Candida* species directly from blood samples of ICU patients meeting specific clinical criteria using multiplex real-time PCR, and to determine the susceptibility profiles of culture-confirmed isolates against key antifungals using the CLSI disc diffusion method.

## Materials and methods

This cross-sectional study was conducted over a period of one year, from September 2022 to August 2023. Clinical samples were obtained from patients admitted to the Intensive Care Unit (ICU) of Bangladesh Medical University (BMU). All microbiological and molecular laboratory procedures were carried out in the Department of Microbiology & Immunology, BMU.

Study population

Patients admitted to the ICU of BMU during the study period, who exhibited clinical suspicion of invasive candidiasis, such as persistent fever, sepsis unresponsive to antibiotics, or risk factors including broad-spectrum antibiotic use, central venous catheters, total parenteral nutrition, recent surgery, or immunosuppression, were included in the study. Only patients from whom appropriate clinical specimens (blood or sterile body fluids) could be collected before initiation of antifungal therapy were eligible. Patients receiving antifungal treatment for more than 48 hours before sample collection, those with contaminated or inadequate samples, and those unwilling to provide consent were excluded. A purposive sampling method was employed to select cases that met the predefined clinical suspicion criteria for invasive candidiasis, ensuring that only individuals with relevant risk factors and clinical presentations were enrolled. This approach facilitated focused sampling of high-risk ICU patients to optimize species detection and antifungal susceptibility assessment.

Sample size

Based on an expected prevalence of invasive candidiasis of 31% among ICU patients in Bangladesh [3] and a 95% confidence interval, a margin of error of approximately 12% was used.

The sample size was calculated using the single population proportion formula:

$n = \frac{Z^{2} (p q)}{d^{2}}$

Where:

n = required sample size

Z = Z-value corresponding to 95% confidence interval (= 1.96)

p = expected prevalence

q= (1-p)

d = margin of error (precision)

This yields a calculated sample size of 57 clinical specimens. All eligible patients meeting the inclusion criteria were enrolled consecutively using purposive sampling until the target sample size was reached. A sample size of 60 is used for this study.

Laboratory procedure

Method of Blood Collection and Processing

Blood samples were collected from patients suspected of invasive fungal infections who had fulfilled the inclusion criteria. All primary blood cultures were done by automated blood culture (BD-BACTEC; Becton Dickinson, NJ, US) methods. With full aseptic precautions, venepuncture sites were disinfected with 70% alcohol followed by 1% tincture of iodine. A total of 16 mL of blood was collected using a sterile syringe and butterfly needle and divided into three parts: 10 mL for blood culture, 3 mL into an ethylenediaminetetraacetic acid (EDTA) vial for DNA work, and 3 mL into a plain tube for serum separation. Samples were transported immediately to the Department of Microbiology and Immunology, BMU, in a leak-proof biohazard container.

For culture processing, the venepuncture needle was replaced with a sterile needle, and the BD-BACTEC™ Plus Aerobic/F bottle cap was disinfected with 70% alcohol before inoculating 10 mL of blood. Bottles were labeled and incubated in the BD-BACTEC™ FX40 system at 37 °C for 1-5 days according to manufacturer guidelines.

Positive bottles were sub-cultured aseptically onto Sabouraud dextrose agar, blood agar, and MacConkey agar, incubated at 37 °C for 24 hours (up to 4-5 days for SDA). Gram staining was performed on the subculture growth. Culture bottles and media were discarded after seven days, following biosafety protocols.

Isolation of *Candida* species

Culture

Colony morphology: On blood agar, the *Candida* species produced cream-colored, pasty, round, moist colonies. They also produced extensions, called ‘feet’, which were developed at the border of the colony.

On Sabouraud agar (SDA) media, the *Candida* species produced cream-colored, pasty, round, moist colonies with a distinct yeast smell.

Microscopic Examination

Wet film: A suspension was prepared from the isolated colony on SDA media, mixing with one drop of sterile normal saline on a glass slide. After placing a cover slip, it was examined using a light microscope under a high-power field.

Interpretation: Isolates of *Candida* species were revealed as oval or round cells ranging from 4 to 6 µm with or without budding.

Gram staining: A smear was prepared on a glass slide by mixing a single yeast colony with one drop of sterile normal saline. Then, Gram staining was performed, and the slide was examined under an oil-immersion lens.

Interpretation: Gram-positive yeast cells with or without budding were found.

Molecular Identification of Candida Species by Real-Time PCR

Detection of Candida species was performed using the Bosphore Candida Basic Panel Kit v2 (Anatolia Geneworks, Turkey), a real-time PCR assay designed to identify and differentiate *C. albicans*, *C. glabrata*, *C. krusei*, and *C. parapsilosis*. PCR amplification was carried out on an ABI 7500 real-time PCR system (Applied Biosystems, USA) following the manufacturer's protocol. The assay employs fluorescence-based detection with internal controls to ensure assay reliability and accuracy. Briefly, colonies were suspended in lysis buffer, subjected to mechanical disruption, and treated with proteinase K for enzymatic digestion. DNA was eluted in 50 µL of nuclease-free water, and purity was confirmed by measuring the A260/A280 ratio using a spectrophotometer [4]. Species-level identification was performed using a multiplex, real-time PCR assay targeting the ITS1-5.8S-ITS2 rDNA region, which allows differentiation of clinically relevant species, including *C. albicans*, *C. tropicalis*, *C. parapsilosis*, *C. glabrata*, and *C. krusei* [3,6]. Primer sequences were not disclosed by the company. Each 25 µL reaction mixture contained SYBR Green master mix, species-specific primers, and 2-5 µL of template DNA. Amplification was performed on a real-time PCR thermocycler under the following cycling conditions: initial denaturation at 95 °C for 5 min, followed by 40 cycles of 95 °C for 15 s and 60 °C for 60 s, with a melt curve analysis at the end to confirm specificity. Positive controls (reference strains) and negative controls (nuclease-free water) were included in each run. Results were interpreted based on threshold cycle (Ct) values and species-specific melt peaks, ensuring accurate detection even in mixed infections [10,11].

Antifungal Susceptibility Testing by Disc Diffusion

The phenotypic antifungal susceptibility of the isolates was assessed using the disc diffusion method according to CLSI M44-A guidelines [7]. Briefly, a 0.5 McFarland yeast suspension was prepared from 24-hour-old colonies grown on SDA and uniformly inoculated onto Mueller-Hinton agar supplemented with 2% glucose and 0.5 µg/mL methylene blue using a sterile swab. Antifungal susceptibility testing was performed using the CLSI M44-A Ed3 guidelines for disk diffusion. The following antifungal agents were tested: fluconazole (25 µg), itraconazole (10 µg), voriconazole (1 µg), and amphotericin B (20 µg). CLSI recommended these agents for the testing of *Candida* species. Plates were incubated at 35 °C for 24 hours, and zones of inhibition were measured in millimeters. Results were interpreted as susceptible (S), susceptible-dose-dependent (SDD), or resistant (R) according to CLSI breakpoints. *C. parapsilosis* ATCC 22019 and *C. krusei* ATCC 6258 were used as quality control.

This method provides a reliable, cost-effective, and widely applicable approach for determining antifungal resistance patterns in clinical isolates [6,8].

Data analysis and management

Data were collected using a pre-designed data collection sheet and subsequently reviewed for adequacy, relevance, and consistency; any irrelevant or inconsistent entries were excluded during quality control. Descriptive statistics, including frequency, percentage, mean, median, standard deviation, and 95% confidence intervals, were generated as appropriate. Data were checked, edited, and analyzed using SPSS version 27 (IBM Corp., Armonk, NY, US).

Ethical considerations

This study was ethically approved by the Institutional Review Board (IRB), BMU (NO. BMU/2022/12411) on 12/12/2022.

## Results

A total of 60 clinical specimens were collected from ICU patients with suspected invasive candidiasis during the study period.

Table 1 shows the age group according to gender distribution in the study population. Out of 60 patients, 5 (13.9%) were male and 1 (4.2%) were in the age group of 18-30 years, 5 (13.9%) were male, and 2 (8.3%) were female in the age group of 31-40 years, 5 (13.9%) were male and 5 (13.9%) were female in the age group of 41-50 years, 9 (25.0%) were male and 5 (13.9%) were female in the age group of 51-60 years, and 12 (33.3%) were male and 11 (45.8%) were female in the age group of ≥60 years. The majority of the cases were from the age group of ≥60 years, male. The mean age for males was 51.97±16.74 years, and for females, it was 57.67±14.79 years.

**Table 1:** Age group according to gender distribution in the study population (n=60)

Age group	Male (n=36)	Female (n=24)
18-30 years	5 (8.3%)	1 (1.7%)
31-40 years	5 (8.3%)	2 (3.4%)
41-50 years	5 (8.3%)	5 (8.3%)
51-60 years	9 (15.0%)	5 (8.3%)
≥61 years	12 (20.0%)	11 (18.3)
Mean age (Years)	51.97±16.74	57.67±14.79

Table 2 shows the distribution of clinical risk factors among the study population. Among the study population of 60, mechanical ventilation, central venous (CV) line, dialysis catheter, urinary catheter, prolonged broad-spectrum antibiotic, total parenteral nutrition (TPN), nasogastric (NG) feeding, and diabetes were in 35 (58.3%), 34 (56.7%), 14 (23.3%), 55 (91.7%), 35 (58.3%), 11 (18.3%), 43 (71.7%) and 19 (31.67%), respectively.

**Table 2:** Distribution of clinical risk factors among the study population (n = 60) CV line: central venous line; TPN: total parenteral nutrition; NG feeding: nasogastric feeding

Clinical Risk Factor	Number of Patients (n)	Percentage (%)
Mechanical ventilation	35	58.3
Central venous (CV) line	34	56.7
Dialysis catheter	14	23.3
Urinary catheter	55	91.7
Prolonged broad-spectrum antibiotics	35	58.3
Total parenteral nutrition (TPN)	11	18.3
Nasogastric (NG) feeding	43	71.7
Diabetes mellitus	19	31.7

The results of real-time PCR for blood fungus are presented in Table 3. Out of 60 blood samples, 38 (63.3%) were positive for real-time PCR. Among the 38 real-time PCR-positive samples, 24 were Candida species. Among those, 8 (33.33%) cases were *C. albicans*, 9 (37.5%) were *C. parapsilosis*, 6 (25%) were *C. glabrata,* and 1 (4.1%) was *C. krusei*. The most commonly identified *Candida* species was *C. parapsilosis*.

**Table 3:** Species of Candida identified by real-time PCR of blood for fungus (n=24) PCR: polymerase chain reaction

PCR	Number (n)	Percentage (%)
Candida albicans	8	33.33
Candida parapsilosis	9	37.5
Candida krusei	1	4.1
Candida glabrata	6	25

Antifungal susceptibility testing by disc diffusion revealed distinct species-specific resistance patterns, as shown in Table 4. Table 4 describes the antifungal susceptibility patterns of different *Candida* species as determined by the disc diffusion method. A total of 24 isolates were tested against four antifungal agents: fluconazole, itraconazole, voriconazole, and amphotericin B. *C. albicans* showed complete (100%) susceptibility to all antifungal drugs. *C. glabrata* demonstrated full susceptibility to itraconazole, voriconazole, and amphotericin B, but only 50% susceptibility to fluconazole. *C. parapsilosis* exhibited lower susceptibility rates across all agents, ranging from 22.22% for amphotericin B to 44.44% for fluconazole and itraconazole. *C. krusei* showed complete resistance to azoles (fluconazole, itraconazole, and voriconazole), while remaining susceptible to amphotericin B. These findings highlight species-specific variations in antifungal susceptibility, emphasizing the importance of accurate identification and susceptibility testing in guiding appropriate therapy.

**Table 4:** Antifungal susceptibility of Candida isolates by disc diffusion method (n=24) S: Susceptible; R: Resistant

Candida species	No. of isolates	Fluconazole	Itraconazole	Voriconazole	Amphotericin B
C. albicans	8	100% S	100% S	100% S	100% S
C. glabrata	6	50% S	100% S	100% S	100% S
C. parapsilosis	9	44.44 S	44.44 S	33.33 S	22.22 S
C. krusei	1	100% R	100% R	100% R	100% S

## Discussion

In the present study, the highest age group was ˃61 years old. This is consistent with the work of Meersseman et al. (2004) and Trelles et al. (2025), where most of the cases of invasive fungal infection tend to occur at high age (older than 60 years), and this can be attributed to the increased incidence of invasive mycoses with bipolarity of age in association with diminished immunity and body resistance [13,14]. On the contrary, Popova and Rogacheva (2025) detected most of the invasive fungal infection cases within the younger age group, as this study was conducted on a patient with hematological malignancy, which shows a higher incidence in this age group [15].

Distribution of risk factors among the studied patients with clinical suspicion of invasive fungal infections (IFIs) revealed a high association of IFIs with urinary catheterization (55; 91.7%) and mechanical ventilation (35; 58.3%). The other risk factors are CV line (34; 56.7%), NG feeding (43; 71.7%), diabetes (19; 31.67%), TPN (11; 18.3%), dialysis catheter (14; 23.3%), and prolonged broad-spectrum antibiotic (35; 58.3%). This result is consistent with a study where the highest percentage of the studied population had urinary catheters, TPN, diabetes, CV line, NG feeding, and prolonged broad-spectrum antibiotics [16]. This result is also consistent with Akbar and Tahawi (2001), where the highest risk factor is associated with urinary catheterization [17].

In our study, *C. albicans* isolates were uniformly susceptible (100%) to all tested antifungal agents (fluconazole, itraconazole, voriconazole, amphotericin B), consistent with multiple reports that *C. albicans *typically retains high susceptibility to azoles and polyenes [18,19]. This result supports the notion that, despite increasing rates of non‑albicans species, *C. albicans* often remains antifungal-sensitive in many clinical settings, though continuous surveillance remains necessary [20].

The pattern observed in *C. glabrata* isolates is particularly noteworthy: while all isolates remained susceptible to itraconazole, voriconazole, and amphotericin B, a significant fraction showed resistance to fluconazole. This mirrors global trends, as *C. glabrata* is known to exhibit reduced susceptibility or intrinsic resistance to fluconazole, making management more challenging [21]. Moreover, disc diffusion has been validated for the rapid detection of fluconazole resistance in *C. glabrata*, with high categorical agreement compared with reference methods [22], suggesting that our methodology is reliable for clinical decision-making.

In *C. parapsilosis*, the mixed susceptibility profile, four isolates sensitive to fluconazole and itraconazole, three resistant to voriconazole, and two resistant to amphotericin B, raises clinical concerns. Non‑albicans species, such as *C. parapsilosis*, are increasingly implicated in invasive infections, and variable azole resistance has been documented in various geographic settings [8]. The resistance to amphotericin B in two isolates is particularly alarming, as this drug is often considered a robust option for serious infections.

The *C. krusei* isolates in our study showed full resistance to all tested azoles but remained susceptible to amphotericin B. This aligns with the well-known intrinsic resistance of *C. krusei* to fluconazole and other azoles, a phenomenon documented in prior studies [8,18]. The maintained sensitivity to amphotericin B underscores its importance as a therapeutic alternative for *C. krusei* infections, especially when azole resistance is present.

Overall, our findings highlight an important epidemiological shift: non‑albicans *Candida* species not only predominate but also exhibit more complex and restrictive susceptibility profiles than *C. albicans*. This has significant implications for empirical therapy in invasive candidiasis, particularly in ICU settings, where delayed or inappropriate antifungal treatment can worsen patient outcomes. The high rate of fluconazole resistance in *C. glabrata* and variable resistance in *C. parapsilosis* suggest that reliance on fluconazole monotherapy may be less effective and riskier. Amphotericin B shows broad activity across species in our study, reinforcing its continued utility, though its known toxicity remains a limitation.

Our results support the urgent need for species-level identification and routine antifungal susceptibility testing in clinical laboratories. Rapid methods, such as disc diffusion, provide practical and cost-effective information that can guide therapy more precisely than empirical treatment. Such targeted therapy may reduce treatment failure, limit the spread of resistant strains, and optimize antifungal stewardship.

This study has several limitations. Due to constraints of time, budget, and resources, a population-based study including a larger number of samples from peripheral settings could not be conducted. Additionally, the unavailability of BD BACTEC-Mycosis IC/F culture bottles necessitated the use of BD BACTEC Plus Aerobic/F bottles, which may have affected fungal recovery rates. Multiple blood cultures, which could have increased the isolation rate of *Candida *species, were also not performed.

Despite these limitations, the findings underscore the utility of species-level identification and antifungal susceptibility testing in critically ill patients. Based on our results, real-time PCR can be recommended as a rapid and reliable diagnostic tool for invasive fungal infections. Future studies should aim to expand the sample size and further improve rapid diagnostic techniques to facilitate the timely initiation of appropriate antifungal therapy, thereby improving patient outcomes.

## Conclusions

This study demonstrates a clear predominance of non-albicans *Candida* species in ICU patients with suspected invasive candidiasis, accompanied by varied and clinically significant antifungal resistance patterns. While *C. albicans* isolates remained uniformly susceptible to all tested antifungals, NAC species, such as *C. glabrata *and *C. parapsilosis*, exhibited reduced susceptibility, particularly to fluconazole, and *C. krusei* showed intrinsic azole resistance. These findings highlight the limitations of empirical fluconazole therapy in ICU settings and underscore the need for species-specific treatment strategies. Real-time PCR proved to be a rapid and reliable tool for accurate detection and differentiation of *Candida* species, enabling early diagnosis compared with conventional methods. Disc diffusion testing further provided practical and cost-effective susceptibility data essential for guiding appropriate therapy. Together, these diagnostic approaches can significantly improve patient management, reduce treatment delays, and support antifungal stewardship.
